# Confirmation of the superior performance of the causal Graphical Analysis Using Genetics (cGAUGE) pipeline in comparison to various competing alternatives

**DOI:** 10.12688/wellcomeopenres.17991.1

**Published:** 2022-07-05

**Authors:** Richard Howey, Heather J. Cordell

**Affiliations:** 1 Population Health Sciences Institute, Faculty of Medical Sciences, Newcastle University, Newcastle upon Tyne, NE1 3BZ, UK

**Keywords:** Causal inference, Mendelian randomization, Bayesian networks

## Abstract

Various methods exist that utilise information from genetic predictors to help identify potential causal relationships between measured biological or clinical traits. Here we conduct computer simulations to investigate the performance of a recently proposed causal Graphical Analysis Using Genetics (cGAUGE) pipeline, used as a precursor to Mendelian randomization analysis, in comparison to our previously proposed Bayesian Network approach for addressing this problem. We use the same simulation (and analysis) code as was used by the developers of cGAUGE, adding in a comparison with the Bayesian Network approach. Overall, we find the optimal method (in terms of giving high power and low false discovery rate) is the cGAUGE pipeline followed by subsequent analysis using the MR-PRESSO Mendelian randomization approach.

## Introduction

In a paper recently published by Amar *et al*. (2021), a pipeline entitled “causal Graphical Analysis Using Genetics” (cGAUGE) was proposed. This pipeline involves carrying out various pre-processing/filtering steps to reduce the number of variables to be taken forward for subsequent causal inference analysis using extensions of Mendelian randomization (MR) approaches, including inverse variance weighted (IVW) regression ( Burgess *et al*., 2013), MR-Egger ( Bowden *et al*., 2015) and MR-PRESSO ( Verbanck *et al*., 2018). Amar *et al*. (2021) demonstrated that use of their cGAUGE pipeline resulted in a lower false discovery rate (FDR) compared to carrying out the same MR analysis approaches with no pre-filtering of variables.

Amar *et al*. (2021) also considered the Bayesian network (BN) approach that we previously described ( Howey *et al*., 2020). However, the BN methods were not optimally implemented by Amar *et al*. (2021), and only a single FDR value (rather than a detailed comparison of FDRs under different scenarios) was reported. We therefore here use the R simulation code of Amar *et al*. (2021) and re-run some of their simulation examples to compare the MR methods (with/without cGAUGE pre-filtering) with our BN approach. We also take the opportunity to evaluate not just the FDR but also the power (at any given FDR) of the different methods considered, which was not reported by Amar *et al*. (2021).

As previously discussed ( Howey *et al*., 2020), there is a lack of direct comparability between MR and BN methods, and so for making discoveries with MR we chose to consider *p*-value threshold values of 0.1, 0.05 and 0.01, while for BN we used edge probability thresholds of 0.7, 0.8 and 0.9. The resulting powers and false discovery rates (FDR) are therefore not directly comparable, but they do give some indication of how the methods perform using thresholds that might be considered reasonably comparable choices in practice. In addition, by plotting receiver operating characteristic (ROC) curves, we can make direct comparisons between the methods in terms of the powers achieved at any given FDR.

## Methods

To reproduce the simulated data and MR analyses of Amar *et al*. (2021), we downloaded the R code they provided. We used it to repeat the results presented in Figure 3a of Amar *et al*. (2021) but we additionally analysed the data using a BN approach. We only considered the “UniqueIV” version of cGAUGE as this had been shown by Amar *et al*. (2021) to perform the best. There were 15 continuous traits simulated, each having between 10 to 20 binary instrumental variables. The traits were set to have random causal relationships with around 1 or 2 other traits. Different levels of horizontal pleiotropy were set through assigning the simulation parameter *p _pleio_* values of 0 *,* 0 *.*1,...0 *.*4, where zero indicates no horizontal pleiotropy. We used 100 simulation replicates and calculated the average false discovery rate (FDR) and the number of causal predictions between variables. We also calculated the average power of each method to identify the true causal relationships in each simulation. For MR, *p*-value threshold values of 0.1, 0.05 and 0.01, adjusted using the BY algorithm ( Benjamini & Hochberg, 1995; Benjamini & Yekutieli, 2001), were used. For BN, edge probability thresholds of 0.7, 0.8 and 0.9 were used.

In our earlier paper ( Howey *et al*., 2020) we described two BN approaches. The first used only four variables (two traits and a weighted allele score variable for each trait) in any given analysis, and the second included all available trait variables simultaneously in the analysis, together with a weighted allele score variable for each trait variable. Here we reproduce Figure 3a of Amar *et al*. (2021), adding in our four-variable BN approach. (This is the approach that was labelled as BN(G,Z) in Figures 3–6 and as B1 in Figure 10 of Howey *et al*. (2020)). Weighted allele score variables were created using genetic instrumental variables (IVs) passing a *p*-value threshold of 0.05 when adjusted with Bonferonni correction from approximately 225 available IVs. We performed four-variable BN analyses for every pair of variables chosen from the 15 trait variables together with their accompanying weighted allele score variables. All variables were treated as continuous. We also separately carried out four-variable BN analyses using weighted allele score variables created from the subset of instrumental variables that were suggested by the “UniqueIV” cGAUGE method. All BN analyses were carried out using our freely available BayesNetty software package.

The rationale for choosing the four-variable BN approach for each trait pair (instead of using all trait variables in one fitted network) was as follows: In the simulated data and related underlying network, Amar *et al*. (2021) considered a variable to be causal on another variable if there was a directed path from one variable to the other with possible intermediate variables. Therefore, when an average BN is fitted to all the data, a fair comparison with the MR methods should account for the many potential paths when calculating the probability of a causal path from one variable to another. There are considerable practical challenges to correctly estimating the probability of a complete causal path from one variable to another from the average networks calculated by the BN approach when all the data is considered. The four-variable BN method avoids all such problems and has the advantage of a simple analysis approach, making it computationally extremely quick, particularly as no random restarts are required in the fitting process.

For the BN analyses we calculated an average network for every pair of trait variables as previously described ( Howey *et al*., 2020). To compute the average network, the data was bootstrapped with replacement 1000 times and the best fit network was fitted at each iteration. The weighted allele score variable for each trait variable was constrained to be fixed as a parent variable and no other edges were permitted to or from the weighted allele score variables. Thus, the only edge in question was between the two traits. The number of times that an edge appeared between two variables in each best fit network was recorded, together with its direction. This allows us to calculate the strength and direction values (between 0 and 1) for each pair of variables, where the strength is defined as the probability (proportion of times) that an edge appears between the two nodes and the direction is the proportion of times that the edge is in a given direction, given that it exists. The overall probability of a directed edge from one variable to another was given by the product of the strength and direction.

We constructed ROC curves by calculating the mean true and false positive rates over the 100 simulation replicates for each test at different ( *p*-value or edge probability) thresholds. Three different levels of horizontal pleiotropy were considered, corresponding to *p _pleio_* parameter values of 0, 0 *.*2 and 0 *.*4, respectively.

## Results and discussion

**Figure 1.**
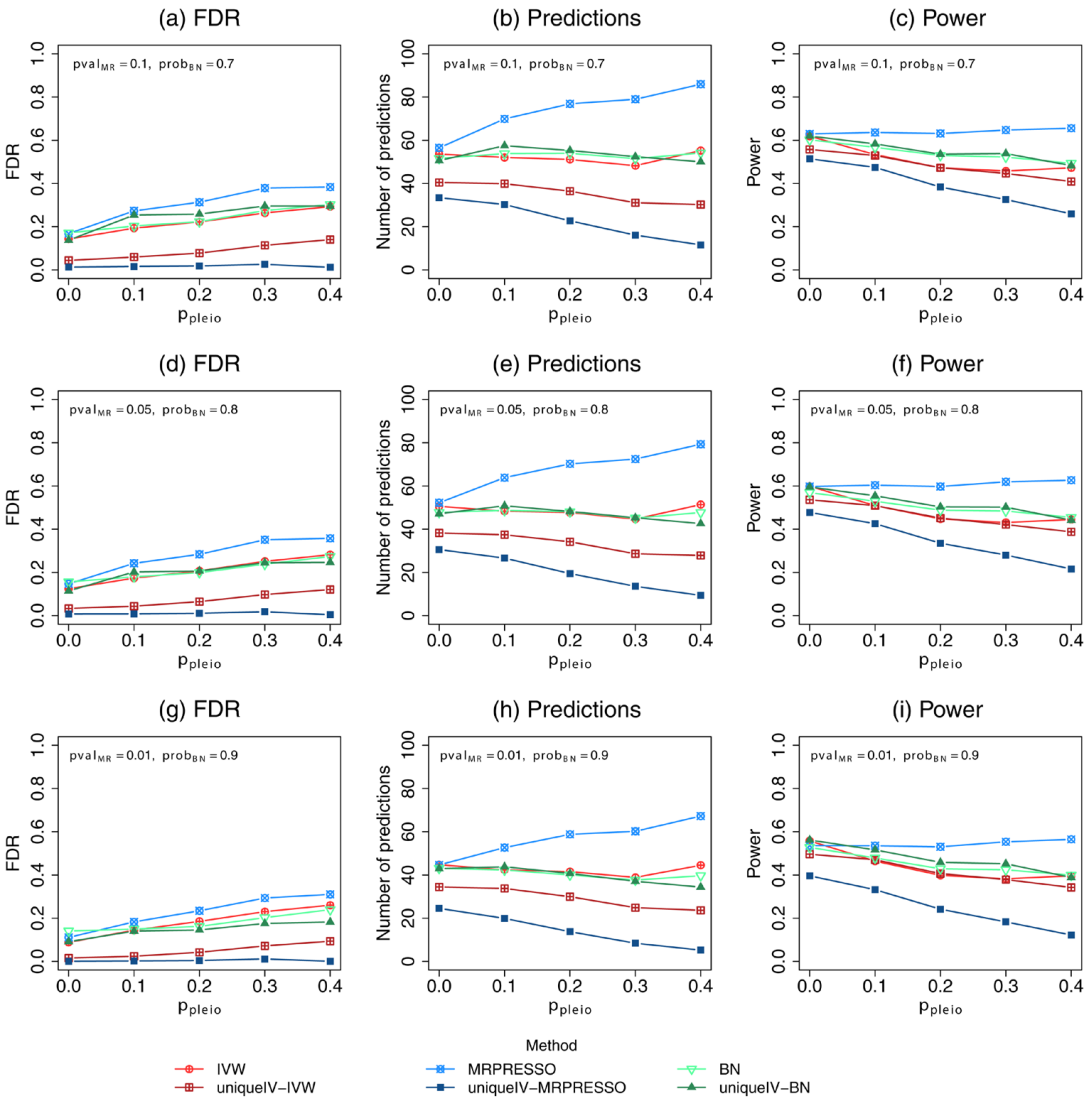
Mean number of discoveries, empirical false discovery rates (FDR) and power of Mendelian randomization and Bayesian network (BN) methods. Data were simulated using the same methods and R code to reproduce plots from Amar *et al*. (2021), with the top left and top middle plots corresponding to Amar *et al*. (2021) Figure 3a. The plots show the average results of the simulations for increasing levels of horizontal pleiotropy given by data simulation parameter *p _pleio_*. Discoveries from each MR test were made at significance levels ( *pval _MR_*) of 0.1, 0.05 and 0.01 in the top, middle and bottom rows respectively, after adjusting for FDR using the BY algorithm. Discoveries from each BN analysis were made at edge probability ( *prob _BN_*) thresholds of 0.7, 0.8 and 0.9 in the top, middle and bottom rows respectively. The columns from left to right show the FDR, number of predictions and the power, respectively. IVW=inverse variance weighted.

Figure 1 shows the average FDR (leftmost panels), number of predictions (middle panels) and power (rightmost panels) for different *p*-value and edge probability thresholds ranging from the least stringent (top panels) to the most stringent (bottom panels). It can be seen by comparing the top left and top middle plots with the top panels of Amar *et al*. (2021) Figure 3a, that we do indeed replicate their results (subject to some random stochasticity). As previously mentioned, the MR FDRs and powers cannot be compared directly with those of BN, but we apply edge probability thresholds that in practice could be considered reasonably similar and we thus consider it reasonable to compare these results.

When *p _pleio_* = 0 there is no horizontal pleiotropy and the FDR for BN is slightly higher than that of other methods, but when *p _pleio_ >* 0 the BN FDR is lower than MR-PRESSO (without use of cGAUGE) and around the same as IVW (without use of cGAUGE). Lower FDRs are achieved by the MR methods when “UniqueIV” cGAUGE filtering is applied, with MR-PRESSO combined with cGAUGE filtering achieving the overall lowest FDR. The number of predictions (middle panels) are included as these were presented in Amar *et al*. (2021), although we consider the power (rightmost panels) to be of more interest. The BN power is reasonably high but decreases when *p _pleio_* increases, similar to what is seen for the MR methods with the exception of MR-PRESSO.

We also tried using the IVs suggested by the cGAUGE method in our BN approach, even though the cGAUGE pipeline was originally designed for use with MR analyses. There was little difference in the FDR or power of BN between using cGAUGE or not. The FDR was around the same, a little higher when the edge probability threshold was 0.7 and a little lower when it was 0.9. The power was negligibly higher for all thresholds. The average FDRs obtained when using BNs with *p _pleio_* = 0 and *p _pleio_* = 0 *.*3 and using an edge probability threshold of 0.7 were 17% and 27%, respectively, similar to those reported by Amar *et al*. (2021). The FDR value for *p _pleio_* = 0 was within the range suggested by Amar *et al*. (2021) (between 16% and 21.4% among the top 20 predicted edges) but for *p _pleio_* = 0 *.*3 the FDR was lower than that suggested by Amar *et al*. (2021)

( *>* 31% among either the top 10 or top 20 predicted edges).

**Figure 2.**
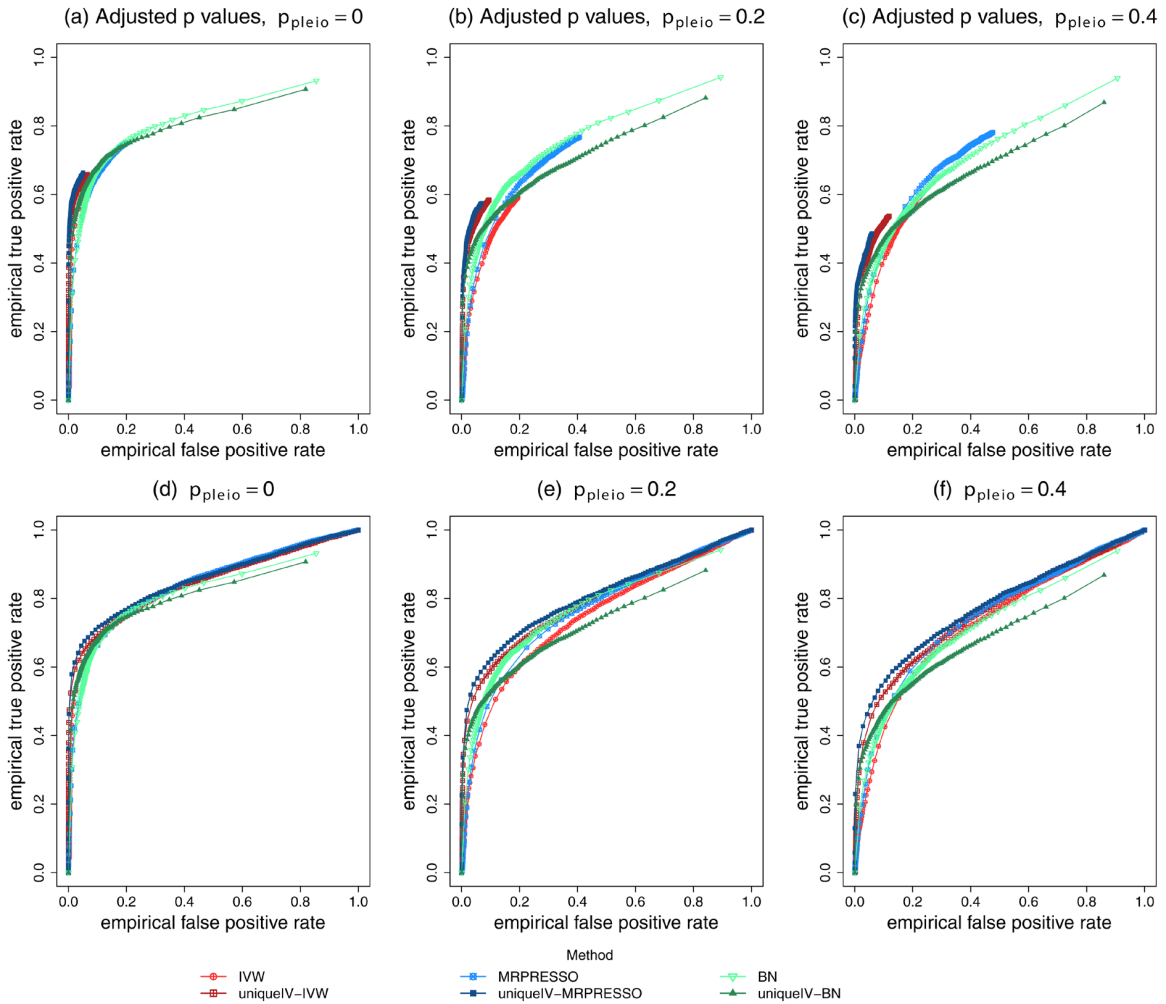
Receiver operating characteristic (ROC) curves of Mendelian randomization and Bayesian network (BN) methods. Data were simulated using the same methods and R code to reproduce the plots of Amar *et al*. (2021) Figure 3a. The ROC curves show results from simulations for different levels of horizontal pleiotropy given by data simulation parameter *p _pleio_* (see Amar *et al*. (2021)) set to either 0, 0.2 or 0.4. The *p*-values of the MR tests were adjusted using the BY algorithm in plots ( **a**) to ( **c**) and not adjusted in plots ( **d**) to ( **f**). IVW=inverse variance weighted.

Figure 2 shows the ROC curves obtained when *p _pleio_* was set to 0, 0 *.*2 and 0 *.*4. The first obvious peculiar feature of the ROC curves in Figure 2 (a) to (c) is that none of the curves reach the top right hand corner (where the true and false positive rates would both equal 1). In BN analysis, on rare occasions the average network may never fit an edge between two traits if there is no strong association, resulting in a zero probability of an edge and thus a true or false positive rate of 1 is not possible for any probability threshold. The results of MR tests are adjusted using the BY algorithm ( Benjamini & Hochberg, 1995; Benjamini & Yekutieli, 2001) using the R package function p.adjust, to be consistent with the results of Amar *et al*. (2021). This adjustment results in many *p*-values that are truncated at 1 and thus do not pass any *p*-value threshold, resulting in no threshold where there is a true or false positive rate of 1. ROC curves in Figure 2 (c) to (d) show the MR methods without any adjustment, which are quite similar to those obtained when adjusting, but extend further to the top right hand corner.

**Figure 3.**
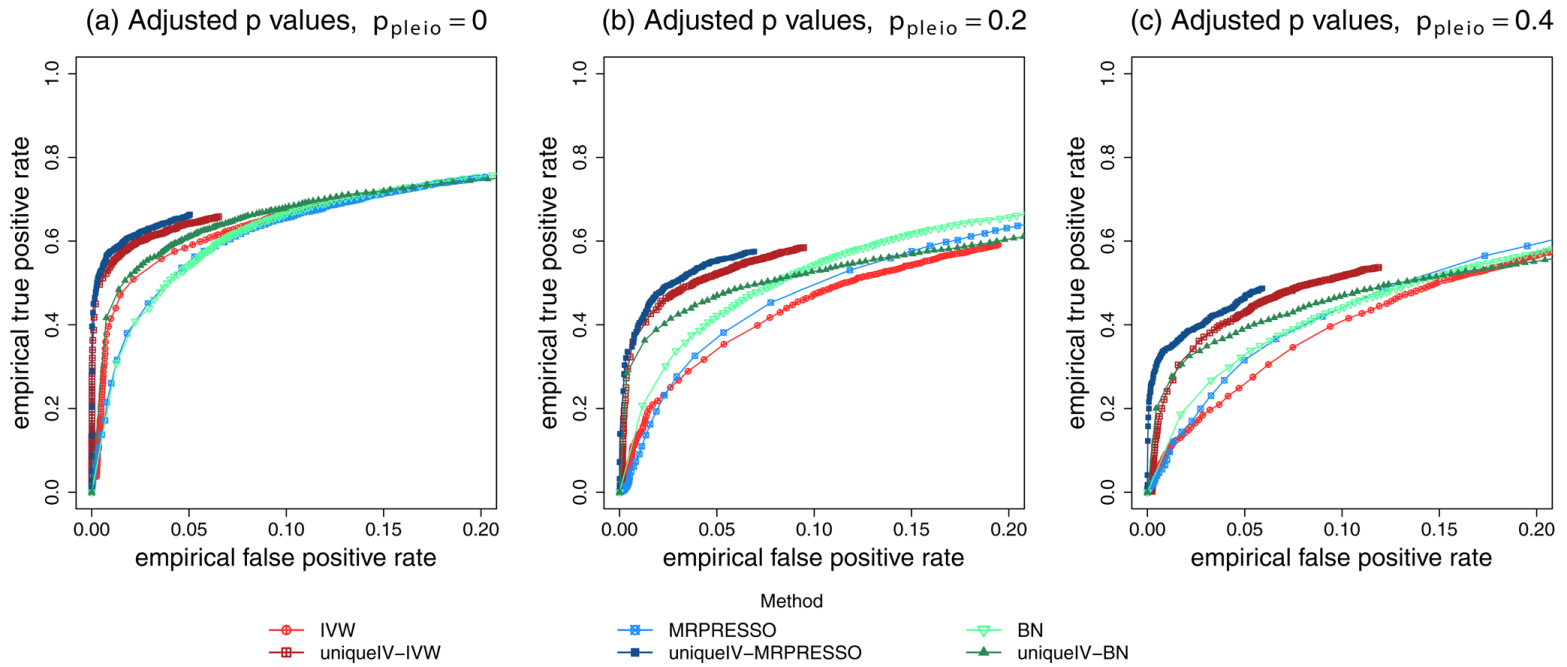
Close-up receiver operating characteristic (ROC) curves of Mendelian randomization and Bayesian network (BN) methods. Data were simulated using the same methods and R code to reproduce the plots of Amar *et al*. (2021) Figure 3a. The ROC curves show results from simulations for different levels of horizontal pleiotropy given by data simulation parameter *p _pleio_* (see Amar *et al*. (2021)) set to either 0, 0.2 or 0.4. The *p*-values of the MR tests were adjusted using the BY algorithm in plots ( **a**) to ( **c**). IVW=inverse variance weighted.

The ROC curves in Figure 2 show the MR methods (particularly MR-PRESSO) that use the cGAUGE pipeline to be better in terms of providing the highest power at any given FDR, especially when there is horizontal pleiotropy, although they do have restricted true positive rates when the *p*-values are adjusted. Figure 3 shows a close-up view of the ROC curves from Figure 2 (focussing on small false positive rates) when the MR *p*-values are adjusted, in which it is easier to observe the overall optimality of the cGAUGE MR methods. When using the cGAUGE IV variables in the BN analyses, it can be seen from Figure 3 that there is an improvement in performance for low false positive rates, but, as shown in Figure 2 and Figure 3, the performance is worse for higher false positive rates.

## Conclusions

In conclusion, our results support the use of the cGAUGE pipeline and suggest that it should ideally be followed by subsequent MR-PRESSO analysis. This approach, at least in the scenarios considered here, generates the highest power at any given FDR, in comparison to competing approaches. However, as previously noted ( Howey *et al*., 2020), BN analysis makes different assumptions from MR and offers a complementary alternative, as MR and BN methods may behave differently when different assumption violations occur. The BN approach has also been extended to incorporate medium-to-large amounts of missing data through a network-informed imputation approach ( Howey *et al*., 2021), which can result in a substantial boost in power when applied to real large-scale data sets.

This research was funded in whole, or in part, by the Wellcome Trust (Grant number 219424/Z/19/Z). For the purpose of open access, the author has applied a CC BY public copyright licence to any Author Accepted Manuscript version arising from this submission.

## Data and software availability

Code to simulate data, along with the R implementation of cGAUGE, were downloaded from https://github.com/david-dd-amar/cGAUGE/. The BayesNetty software package is available from https://www.staff.ncl.ac.uk/richard.howey/bayesnetty/.
