# Supplementary material for: Confirmation of the superior performance of the causal Graphical Analysis Using Genetics (cGAUGE) pipeline in comparison to various competing alternatives
Source: Wellcome Open Res. 2022 Jul 5;7:180. [Version 1] doi: 10.12688/wellcomeopenres.17991.1 (PMC9411974; doi:10.12688/wellcomeopenres.17991.1)
Supplement: Peer review history

# Peer review: Reviewer response for version 1

**DOI:** 10.21956/wellcomeopenres.19948.r51547

**Published:** 2022-08-25

**Authors:** David Amar

**Affiliations:** 1 Division of Cardiovascular Medicine, Stanford University, Stanford, CA, USA

In this paper, Howey and Cordell compare Mendelian Randomization (MR) -based methods, with and without the cGAUGE preprocessing pipeline, to their previously described Bayesian Network (BN) learning approach. The authors first replicate the simulation analysis done by Amar *et al.* 2021, and then extend the discussion by (1) improving the BN learning framework as compared to the implementation in Amar et al., and (2) presenting power and ROC calculations. The authors confirm the top performance of using cGAUGE with MR methods, but also provide further insights on when BNs perform well and how these methods are complementary.

 The paper is well written with detailed explanations of the analysis motivation and parameters. The problem of assessing causal inference methods is important, especially as some methods were repeatedly shown to have low performance (e.g., as shown by Amar et al. and others with respect to MR-Egger). This paper thus contributes by improving and extending the Amar *et al*. study, and by thoroughly discussing how to apply BN methods.

 I only have a few minor comments, as I list below: 

1. Typo: “using the R package function p.adjust” - > ‘“using the R function p.adjust”
2. In the Conclusions section the authors nicely describe how MR and BN are complementary and also discuss their recent method for analysis of real data with missing values. These two points further strengthen the discussion about the utility of BN methods. These points can also be made in the abstract and the introduction to better orient the reader. For example, the authors can mention in the abstract that they also discuss how BNs offer a complementary alternative to MR as these approaches may behave differently when different assumption violations occur.

Is the work clearly and accurately presented and does it cite the current literature?

Yes

If applicable, is the statistical analysis and its interpretation appropriate?

Yes

Are all the source data underlying the results available to ensure full reproducibility?

Yes

Is the study design appropriate and is the work technically sound?

Yes

Are the conclusions drawn adequately supported by the results?

Yes

Are sufficient details of methods and analysis provided to allow replication by others?

Yes

Reviewer Expertise:

Computational Biology, Causality, Machine learning

I confirm that I have read this submission and believe that I have an appropriate level of expertise to confirm that it is of an acceptable scientific standard.

# Peer review: Reviewer response for version 1

**DOI:** 10.21956/wellcomeopenres.19948.r51546

**Published:** 2022-08-10

**Authors:** Nastasiya Grinberg

**Affiliations:** 1 National Institute of Agricultural Botany, Cambridge, UK

The manuscript by Howey & Cordell compares Graphical Analysis Using Genetics (cGAUGE) of Amar et al (2021) ^1^ coupled with various Mendelian Randomisation (MR) techniques with an alternative approach of Bayesian network (BN) of Howey et al (2020). ^2^ cGAUGE is a preprocessing framework to prepare data for the subsequent causal inference analysis, e.g. Mendelian randomization (MR) (which is used to establish causal links between various traits and factors). Authors intend a more thorough comparison of cGAUGE + various MR methods and BN, taking more care implementing BN than Amar et al did. Additionally, power of various methods is calculated.

 Howey & Cordell confirm that preceding MR analysis with cGAUGE yields lower FDR and that cGAUGE followed MR-PRESSO yields the optimal method. They, however, stress that direct comparison of MR and BN is non-trivial and that the two methods make different assumptions.

 The paper is well written and is thorough in describing implementation of the experiment, parameters and settings used and reasons behind their choices, code is available in a GitHub repository. Motivation and conclusions of the work are clearly stated and well discussed. It is a useful piece of work for people intending to use cGAUGE or BN for causal inference analysis.

 If I have to point out one thing it would be lack of comment/discussion of results from the point of view of the number of predictions and power --- whilst FDR results are extensively discussed, the other two measures of performance are not commented upon. Furthermore, it would be helpful to explain what one expects to observe in way of number of predictions --- wouldn't proportion of correctly identified causal links be a better measure?

Is the work clearly and accurately presented and does it cite the current literature?

Yes

If applicable, is the statistical analysis and its interpretation appropriate?

Yes

Are all the source data underlying the results available to ensure full reproducibility?

Yes

Is the study design appropriate and is the work technically sound?

Yes

Are the conclusions drawn adequately supported by the results?

Yes

Are sufficient details of methods and analysis provided to allow replication by others?

Yes

Reviewer Expertise:

machine learning, logic programming, statistics, genomics, quant gen

I confirm that I have read this submission and believe that I have an appropriate level of expertise to confirm that it is of an acceptable scientific standard.

## References

[1] : Graphical analysis for phenome-wide causal discovery in genotyped population-scale biobanks. *Nature Communications* .2021;12(1) : 10.1038/s41467-020-20516-2 10.1038/s41467-020-20516-2 PMC780664733441555

[2] : Bayesian network analysis incorporating genetic anchors complements conventional Mendelian randomization approaches for exploratory analysis of causal relationships in complex data. *PLoS Genet* .16(3) : 10.1371/journal.pgen.1008198 e1008198 10.1371/journal.pgen.1008198 32119656PMC7067488
